# Supplementary material for: Characterization and Expression of TGF-β Proteins and Receptor in Sea Cucumber (Holothuria scabra): Insights into Potential Applications via Molecular Docking Predictions
Source: Int J Mol Sci. 2025 Jul 21;26(14):6998. doi: 10.3390/ijms26146998 (PMC12295056; doi:10.3390/ijms26146998)

International Journal of *Molecular Sciences*

MDPI

Article

Characterization and Expression of TGF-β Proteins and Receptor in Sea Cucumber (*Holothuria scabra*): Insights into Potential Applications via Molecular Docking Predictions

Siriporn Nonkhwao[1], Jarupa Charoenrit[1], Chanachon Ratanamungklanon[1], Lanlalin Sojikul[1], Supawadee Duangprom[1], Sineenart Songkoomkrong[1], Jirawat Saetan[2], Nipawan Nuemket[1], Prateep Amonruttanapun[1], Prasert Sobhon[3] and Napamanee Kornthong[1]*

1. Chulabhorn International College of Medicine, Thammasat University, Rangsit Campus, Pathumthani, Thailand
2. Division of Health and Applied Sciences, Faculty of Science, Prince of Songkla University, Hat Yai, Songkhla, Thailand
3. Department of Anatomy, Faculty of Science, Mahidol University, Bangkok, Thailand

* Correspondence: Napamanee Kornthong, napamaneenatt@gmail.com

Supplementary Data S1.

Nucleotide sequence of *H. scabra* transforming growth factor-β receptor with NCBI's accession numbers PV173756

>PV173756 [organism=*Holothuria scabra*] transforming growth factor-β receptor, 1850 bp DNA

GAGCAGCCCATGAATGTTGGTCCATGTACTAGCTACGATGAGAGATATTACTACGACTTAACCAAGA
TGCTTGCTACCCTTTCCAGTTTGGAGGATGCGAAGATAATGACAACCACTTCCTCACTGAAGAAGCCT
GTCTAGATACTTGCAAAGGTTATAAAAGGGGGAGAGAGCCAACAATGATTGAAATGGCGACACTTA
TTGCCAGTCCTGTCTGTGTCATATGCATCGCTGTAACTCTGTATGTGATTCTACAACACAAGTGTAGAA
AGATGCCTCCGTACCTTCCTACAGATATACAGGACCCCTCATATCCCCATCATCATGAATGTGGCCCT
AATGTCCTTGTGGAGCTCATAAACAAGGAAGGCACTGGTTCTGGCTCTGGTTTACCACTTTTAGTGCA
AAGAACCATTGCTCGACAGATCATACTCCTTGACACGATAGGAAAAGGCCGCTATGGTGAAGTCTAT
AAAGGAAGGTGGAGAGGGGAGTATGTCGCAGTCAAGATATTTTCATCACGGGAGGAGAGATCCTGG
TTCAGAGAGACAGAGATATATCAAACTGTGATGTTGCGACATGCTAACATCCTTGGATTTATTGCTGC
TGATAACAAAGATAATGGTCTCTGCACTCAACTACTTCTGATCACCGACTTCCACGAGCGGGGCTCAC
TCTTTGACTACTTGGACCGTAACACTGTGGATATCCAAGGGATGCTCACACTTTCCCTCTCGTTGGCAA
CAGGTCTTGCTCACCTTCATATGGAAATAGTTGGAATGCAAGGTAAACCCGCCATAGCTCATAGAGA
TCTCAAAAGCAAAAATGTTCTCGTCAAAGGGAACGGTCAGTGTGCCATTGCTGACCTTGGCCTGGCA
GTGAGGCACATTTCAGCCACTGACACGGTGGACATTGCCCAGAACAACAGAATTGGTACAAAGAGG
TACATGGCACCTGAGGTGCTGAATGACACCCTCAATCGAAATCACTTTGACTCTTTCAAGAGAGCAG
ATATTTATTCACTTGGACTTATTCTTTGGGAAATAGCAAGGAGATGCTGTGTTGGAGGTATATACGAA
GAATATCACTTGCCATACTATGACATGGTGCCAACAGATCCATCCCTGGATGAGATGAGGAGAGTTG
TATGCTTAGAGAACAGACGCCCATCCATACCCAATCGCTGGAATAGCTATGAGGAATTACGGGTGAT
AGCAAAGATTATGAAGGAATGCTGGTATGCCAACGGAGGAGCTCGTCTCACAGCCCTGCGAATCAA
GAAAACTCTGACTACCCTTCATCCATTGGAGGATATTAAGGGATGAAGTATCTGTTGAGTTCATTGTA
TGTTATCATGCTACGAACCTTCTTTGGCTGTTTTCTCATTAAAATTCATACCTTCGGTACGTCCTGGTCG

AAAGTTGGACCCCATCACAAGGAGCTGAAGGCGGTGTACCTTGAACAAGGGTGTTTGCTGACCCAG
GGATCGTGCTGCTTTCACAAGGTATTGGTGGTCTATGGATTCTAGATTGGAGGTCGTGAAGCACCATT
TTTTCAAAGACACTCTATTTGTGGTGAAGTCACCGAGTACAGCACTGAATAAGAAATAAAAAACCTC
TTTTTTTACAAATACATGTATATTGAAAATTATCAGTGGTGATGTGTTTGTGATGATGGTGTACATGTA
AACTGCCCGCTCTATTTCATGTAAGCCGTTCTTTCCAGTTGGGACTTACGCCACTCTTCTTCTGTAAAT
GTATGTAATATTGAAAAAATTTATGCCGGAAGAGCCACTTGGAATTGGTTTTCATTCATGTATCTTGC
AGCACACTAAAAAGATGATCTGGT

Supplementary gel electrophoresis

Agarose gel electrophoresis of PCR product using specific primer of HolscTGFBR (207 bp). (A) Tissue-specific expression of HolscTGFBR gene, and (B) the 16s rRNA expression was used as a positive control. Lane 1: 100 bp DNA marker, Lane 2: RNC, radial nerve cord, Lane 3: NR, nerve ring, Lane 4: IW, inner wall, Lane 5: TT, testis, Lane 6: OV, ovary, Lane 7: RT, respiratory tree, Lane 8: IN, intestine, Lane 9: LM, longitudinal mussel, Lane 10: N, non-template (negative control).

(A)

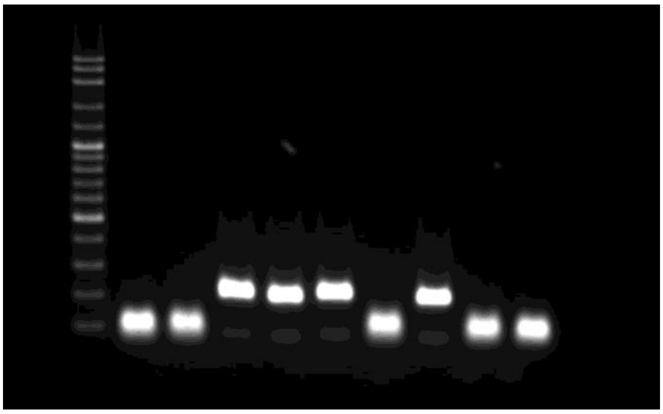

(B)

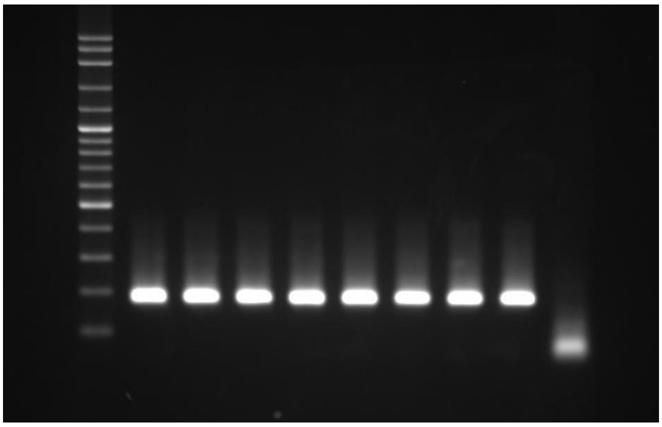

Supplement: Supplementary file 1 [file ijms-26-06998-s001.zip › ijms-3729725-supplementary.pdf]
